# Unique α-synuclein pathology within the amygdala in Lewy body dementia: implications for disease initiation and progression

**DOI:** 10.1186/s40478-019-0787-2

**Published:** 2019-09-02

**Authors:** Zachary A. Sorrentino, Marshall S. Goodwin, Cara J. Riffe, Jess-Karan S. Dhillon, Yuxing Xia, Kimberly-Marie Gorion, Niran Vijayaraghavan, Karen N. McFarland, Lawrence I. Golbe, Anthony T. Yachnis, Benoit I. Giasson

**Affiliations:** 1 Department of Neuroscience, College of Medicine, University of Florida, Gainesville, FL 32610 USA; 2 Center for Translational Research in Neurodegenerative Disease, College of Medicine, University of Florida, Gainesville, FL 32610 USA; 3 Department of Neurology, College of Medicine, University of Florida, Gainesville, FL 32610 USA; 4 McKnight Brain Institute, College of Medicine University of Florida, Gainesville, FL 32610 USA; 5 Rutgers Robert Wood Johnson Medical School, New Brunswick, NJ 08901 USA; 6 Department of Pathology, College of Medicine, University of Florida, Gainesville, FL 32610 USA

**Keywords:** α-Synuclein, Parkinson’s disease, Lewy body dementia, Truncation, Amygdala, Inclusion formation, Neurodegeneration, Astrocyte, Lewy body

## Abstract

The protein α-synuclein (αsyn) forms pathologic aggregates in a number of neurodegenerative diseases including Lewy body dementia (LBD) and Parkinson’s disease (PD). It is unclear why diseases such as LBD may develop widespread αsyn pathology, while in Alzheimer’s disease with amygdala restricted Lewy bodies (AD/ALB) the αsyn aggregates remain localized. The amygdala contains αsyn aggregates in both LBD and in AD/ALB; to understand why αsyn pathology continues to progress in LBD but not in AD/ALB, tissue from the amygdala and other regions were obtained from 14 cases of LBD, 9 cases of AD/ALB, and 4 controls for immunohistochemical and biochemical characterization. Utilizing a panel of previously characterized αsyn antibodies, numerous unique pathologies differentiating LBD and AD/ALB were revealed; particularly the presence of dense neuropil αsyn aggregates, astrocytic αsyn, and αsyn-containing dystrophic neurites within senile plaques. Within LBD, these unique pathologies were predominantly present within the amygdala. Biochemically, the amygdala in LBD prominently contained specific carboxy-truncated forms of αsyn which are highly prone to aggregate, suggesting that the amygdala may be prone to initiate development of αsyn pathology. Similar to carboxy-truncated αsyn, it was demonstrated herein that the presence of aggregation prone A53T αsyn is sufficient to drive misfolding of wild-type αsyn in human disease. Overall, this study identifies within the amygdala in LBD the presence of unique strain-like variation in αsyn pathology that may be a determinant of disease progression.

**Electronic supplementary material:**

The online version of this article (10.1186/s40478-019-0787-2) contains supplementary material, which is available to authorized users.

## Introduction

Aggregates comprised of the pre-synaptic neuronal protein, α-synuclein (αsyn), are the major component in Lewy body (LB) inclusions that pathologically define Parkinson’s disease (PD) and Lewy body dementia (LBD) [52, 96]; additionally 40–60% of Alzheimer’s disease (AD) cases display LBs that are most commonly localized to the amygdala [3, 37, 73, 84, 102]. αsyn aggregates are not merely associated with these diseases but in fact can have etiologic roles, whereby aggregation promoting point mutations (A53T in particular) in the *SNCA* gene encoding αsyn have been discovered to cause familial PD/LBD [15, 20, 83]. It is unclear what factors prompt physiologic αsyn to misfold and form pathologic inclusions, however once formed these aggregates are key to disease progression as they can likely spread between cells and induce further pathology along with resultant cellular toxicity in a prion-like fashion [21, 54, 103, 110]. Prominent in synucleinopathies is the occurrence of post-translational modifications of αsyn which may influence the tendency of the protein to misfold and aggregate; in disease, 90% or more of αsyn becomes phosphorylated at Ser129 and 10–20% may become carboxy (C)-terminally truncated within LB enriched extracts [2, 4, 53, 60, 62]. C-terminal truncation of αsyn in particular may be crucial, as these species aggregate even more readily than disease-causal mutant forms of αsyn [16, 40, 41, 71, 72, 90, 93]. Another important modulator of αsyn pathology in LBD and AD is concurrent AD pathologic changes such as tau neurofibrillary tangles and Aβ plaques which are present at a moderate to severe stage in the majority of LBD cases and worsen clinical outcomes [43, 44, 97, 100]. Tau and Aβ purportedly harbor prion-like properties similarly to αsyn and have in-vitro demonstrated the capacity to cross-seed αsyn aggregation [32, 38, 77, 95] which may be evidenced in human disease by lesions containing both misfolded tau and αsyn within the same cell; these co-localized aggregates are often within the medial temporal lobe (MTL) of LBD patients [33, 46, 49, 88].

The prion-like spread model of αsyn pathology is complicated due to the presence of atypical synucleinopathies that do not conform to typical staging schema of caudal to rostral spread [11, 39, 48, 85, 100]; in particular, AD with amygdala predominant LB pathology (AD/ALB) is especially confounding as extensive αsyn aggregates may be predominantly present within just one brain region and lack evidence of initial pathology elsewhere or continued spread to other regions [37, 100, 102]. Furthermore, different synucleinopathies appear to have separate patterns of regional initiation and progression of pathology; αsyn aggregation in the brain purportedly begins within autonomic medullary neurons in PD temporally succeeded by mesencephalic and telencephalic regions which contrasts with LBD in which olfactory and limbic structures may display extensive pathology with minimal midbrain involvement [10, 48, 49, 52, 100, 112]. In addition to heterogeneity in pathologic progression, αsyn aggregates can appear in a number of different morphologies in the same brain including Lewy neurites (LNs), cortical LBs, classical brainstem LBs with a clearly defined core and halo, and other forms often distinct across anatomic regions [24, 36, 49, 105]. Separate types of Lewy pathology may correlate with symptomatic progression differentially; for example, it has been suggested that small, neuritic aggregates of αsyn present in the cortex predict symptomatic severity superiorly to the presence of cortical LBs alone [14, 59, 89]. Similarly, total burden of Lewy pathology within the SNpc to include smaller neuropil aggregates predicted the degree of striatal dopaminergic deficit whereas counts of only brainstem type LBs did not correlate with severity of dopaminergic loss [57]. The diversity of pathologic presentations of synucleinopathies both between and within disease types has been noted extensively in support of the notion that different “strains” of pathologic αsyn may occur [23, 79, 81].

If the prion-like hypothesis of αsyn aggregation holds true, then therapeutic approaches should focus on brain regions not only in which aggregates initially form, but as well those in which more fulminant “strains” occur which may be evidenced by biochemically and histochemically unique properties of αsyn pathologies [23, 26, 80]. The amygdala is an optimal brain region to study in relation to factors governing the transition of LB pathology from an incidental and localized finding as is the case in AD/ALB [74, 86, 102] and possibly incidental Lewy body disease (iLBD) [25, 30, 64] versus a toxic, seemingly prion-like neurodegenerative disorder in LBD and PD. Furthermore, the amygdala is uniquely vulnerable in a number of neurodegenerative diseases as it is afflicted early on in multiple disorders and prone to develop concurrent pathologies [73]. This study aims to define the biochemical and immunohistochemical differences in the intrinsic nature of Lewy related pathology (LRP) within the amygdala in LBD compared with AD/ALB to reveal the molecular alterations in pathologic αsyn aggregates associated with pathology that remains localized in AD/ALB but not LBD. Additionally, the LBD amygdala was compared with other brain regions in LBD to probe for differences in pathology that may underlie the early involvement of the amygdala compared to the midbrain and higher cortical regions.

## Materials and methods

### Autopsy case material

Human brain tissue was obtained through the University of Florida Neuromedicine Human Brain Tissue Bank in accordance with institutional review board approval. Post-mortem pathological staging and diagnoses were made according to respective neuropathological criteria for AD and LBD [42, 70]. Apolipoprotein E (APOE) genotypes were determined by genotyping SNPs rs7412 and rs429358 using TaqMan SNP genotyping assay (ThermoFisher). For immunohistochemical studies, sections from the cingulate cortex, amygdala, and midbrain of 9 formalin fixed cases of diffuse LBD and 9 formalin fixed cases of AD/ALB were used. Some studies included midbrain and hippocampus sections from a previously described patient with the SNCA A53T mutation [27]. For biochemical fractionation and comparison, frozen brain tissues from the medial temporal lobe, amygdala, or cingulate cortex grey matter from 8 cases of diffuse LBD, 2 cases of AD/ALB, and 4 controls were used. In total, the case selection includes 14 sporadic LBD, 1 familial PD/LBD, 9 AD/ALB, and 4 controls without synucleinopathy (Table 1).

**Table 1:** Autopsy case demographics

	Age at Onset	Age at Death	Pathology Diagnosis	APOE	Braak Stage	Thal Phase	CERAD Score	IHC	Biochem
LBD									
Case 1	71	81	LBD/AD/CAA	ε3/ε3	V-VI	5	C3	A, C, M	–
Case 2	60	68	LBD/AD/CAA	ε3/ε4	VI	5	C3	A, C, M	C
Case 3	70	83	LBD/AD	ε3/ε4	VI	5	C3	A, C, M	–
Case 4	70	80	LBD/AD	ε3/ε3	III	3	C2	A, C, M	–
Case 5	73	80	LBD/AD	ε3/ε4	VI	5	C3	A, C, M	MTL
Case 6	51	62	LBD/AD	ε3/ε3	II	5	C3	A, C, M	–
Case 7	63	67	LBD/AD	ε3/ε4	III	4	C3	A, C, M	A, MTL
Case 8	62	67	LBD/AD	ε3/ε4	III	3	C1	A, C, M	–
Case 9	84	90	LBD/AD	ε3/ε3	III	5	C2	A, C, M	–
Case 10	67	72	LBD/AD	ε2/ε3	V-VI	3	C2	–	C
Case 11	59	66	LBD/AD/CAA	ε3/ε4	IV	2	C3	–	C
Case 12	40	78	LBD/AD/CAA	ε3/ε3	IV	3	C3	–	MTL
Case 13	64	74	LBD/AD/CAA	ε2/ε4	VI	5	C2	–	MTL
Case 14	62	68	LBD/AD	ε4/ε4	V	3	C3	–	MTL
AD/ALB									
Case 15	(> 90)	(> 90)	AD/CAA	ε2/ε3	V-VI	3	C3	A, C, M	–
Case 16	–	82	AD	ε2/ε3	III	3	C1	A, C, M	–
Case 17	58	63	AD	ε3/ε3	VI	3	C3	A, C, M	–
Case 18	75	83	AD/CAA	ε3/ε4	V	3	C2	A, C, M	–
Case 19	62	77	AD/PSP	–	II	3	C2	A, C, M	–
Case 20	57	64	AD/CAA	ε3/ε3	V	5	C3	A, C, M	MTL
Case 21	40	77	AD/CAA	ε2/ε3	II	2	C2	A, C, M	MTL
Case 22	(> 90)	(> 90)	AD/CAA	ε3/ε3	VI	4	C3	A, C, M	–
Case 23	53	67	AD	ε3/ε3	VI	3	C3	A, C, M	–
Controls									
Case 24	–	87	No neurological diagnosis	ε2/ε3	0	0	0	–	MTL
Case 25	56	67	FTLD-TDP	ε3/ε3	0	0	0	–	MTL
Case 26	–	82	Cerebrovascular arteriolosclerosis	ε2/ε3	II	2	C1	–	C
Case 27	–	52	No neurological diagnosis	ε3/ε4	II	2	C1	–	C

*LBD* Lewy body dementia, *AD* Alzheimer’s disease, *AD/ALB* Alzheimer’s disease with Amygdala restricted Lewy bodies, *CAA* cerebral amyloid angiopathy, *PSP* progressive supranuclear palsy, *FTLD-TDP* frontotemporal lobar degeneration with TAR DNA-binding protein 43 inclusions, *APOE* apolipoprotein E, *A* amygdala, *C* cingulate cortex, *M* midbrain, *MTL* medial temporal lobe

### Transgenic mouse tissue

Hemizygous M83 transgenic mice overexpress human αsyn harboring the A53T mutation and when intra-muscularly seeded with pre-formed αsyn fibrils they accumulate pathologic inclusions that spread throughout most of the neuro-axis [94]. Hemizygous M20 mice overexpress wild type (WT) human αsyn and when intra-cerebrally injected with preformed αsyn fibrils develop extensive αsyn pathology [91]. Paraffin embedded sections from the brains of the M20 and M83 mouse lines, induced to develop pathology, were obtained in order to confirm the specificity of antibody 3H11.

### Antibodies

Anti-phosphorylated Ser129 (pSer129) αsyn rabbit monoclonal antibody EP1536Y was obtained from Abcam (Cambridge, MA). Antibodies 9C10 and 94-3A10 are mouse monoclonal antibodies specific for N-terminal (2–21) or C-terminal (130–140) residues of αsyn respectively [22]. Antibody 3H11 is a mouse monoclonal antibody raised against central residues (43–63) of human αsyn that does not react with αsyn harboring the A53T mutation [23, 92]. Antibody 5G4, a mouse monoclonal antibody raised against central residues (44–57) with high affinity for oligomeric αsyn, was obtained from MilliporeSigma (Burlington, MA, USA) [56, 58]. Antibody 7F2 is a mouse monoclonal antibody generated against the AT8 epitope specific for phosphorylated tau particularly at pT205 [98]. Antibody 33.1.1 is a mouse monoclonal antibody raised against Aβ residues 1–16 that detects Aβ plaques [69]. Other antibodies utilized include polyclonal rabbit anti-glial fibrillary acidic protein (GFAP) from Dako (Santa Clara, CA, USA) and polyclonal rabbit anti-vimentin (C-20) from Santa Cruz (Dallas, TX, USA).

### Expression and purification of recombinant αsyn proteins

Recombinant WT or A53T human αsyn were expressed from the pRK172 plasmid containing the cDNA for the *SNCA* gene as described previously [34, 107]. Constructs were expressed in *E.coli* BL21 (DE3) and purified as previously described utilizing size exclusion and Mono Q anion exchange chromatography [34]. Recombinant proteins were diluted in pH 7.4 sterile phosphate buffered saline (PBS)(Invitrogen), and concentrations were determined using the bicinchoninic acid assay (BCA) from Pierce (Waltham, MA, USA) with bovine serum albumin (BSA) as the standard.

### Biochemical fractionation

Unfixed frozen human tissue was thawed and samples were retrieved from grey matter of the hippocampus and surrounding MTL structures; when identified, the amygdala was separately processed. For all cases, ~ 250 mg of MTL or amygdala was homogenized within 3 mL/g tissue high-salt (HS) buffer (50 mM Tris–HCl, pH 7.5, 0.75 M NaCl, 2 mM EDTA, 50 mM NaF with a cocktail of protease inhibitors) and sedimented at 100,000 x g for 30 min at 4 °C. The HS supernatant was collected (HS fraction) and insoluble material was re-suspended in 2 mL/g tissue HS buffer/1% Triton X-100. Re-suspended material was again sedimented at 100,000 x g for 30 min at 4 °C and the supernatant was collected (HS/T fraction). Pellets were homogenized in 3 mL/g tissue HS buffer/1 M sucrose and sedimented at 100,000 x g for 30 min at 4 °C in order to remove myelin which was discarded in the supernatant. Pellets were subsequently re-suspended in 2 mL/g tissue radioimmunoprecipitation assay (RIPA) buffer (50 mM Tris, pH 8.0, 150 mM NaCl, 5 mM EDTA, 1% NP-40, 0.5% sodium deoxycholate, 0.1% SDS) and sedimented at 100,000 x g for 30 min at 4 °C; supernatants were collected as the RIPA fractions. Lastly, remaining insoluble material was homogenized in 1 mL/g tissue SDS/Urea buffer (4 M urea, 2% SDS, 25 mM Tris–HCl pH 7.6), probe sonicated, and then stored as the SDS/Urea fraction. The concentration of each fraction was determined using the BCA assay with BSA as the standard. SDS containing sample buffer was added to sequential fractions and all samples were further boiled for 10 min except for the SDS/Urea fraction. Cingulate grey matter was previously obtained and fractionated similarly [23]. Fractions were stored at − 80 °C until western blot analysis.

### Immunohistochemistry

Immunostaining of the sections was performed using established methods [26]. Paraffinized sections were rehydrated and subsequent antigen retrieval was performed in a steam bath for 60 min in a solution of modified citrate buffer (Target Retrieval Solution Citrate pH 6; Agilent, Santa Clara, CA). If indicated, additional antigen retrieval was performed by exposing sections to 70% formic acid for 20 min at room temperature. Endogenous peroxidase was quenched by incubation of sections in 1.5% hydrogen peroxide/0.005% Triton-X-100/PBS solution for 20 min. Non-specific antibody binding was minimized with a 2% fetal bovine serum (FBS)/0.1 M Tris, pH 7.6 block solution; primary antibodies were diluted in block solution and applied to tissue sections at 4 °C overnight. A mixture of biotinylated secondary antibody (Vector Laboratories; Burlingame, CA) and Impress polymer secondary antibody (Vector Laboratories; Burlingame, CA) were similarly diluted in block solution and applied to sections for 1 h at room temperature. An avidin-biotin complex (ABC) system (Vectastain ABC Elite kit; Vector Laboratories, Burlingame, CA) was used to enhance detection of the immunocomplexes, which were visualized using the chromogen 3,3′-diaminobenzidine (DAB kit; KPL, Gaithersburg, MD). Tissue sections were counterstained with hematoxylin. Slides were digitally scanned using an Aperio ScanScope CS instrument (40× magnification; Aperio Technologies Inc., Vista, CA), and images of representative areas of pathology were captured using the ImageScope software (40× magnification; Aperio Technologies Inc.). Semi quantitative assessment of LRP was performed by two independent observers.

### Immunofluorescence

Deparaffinization and antigen retrieval procedures were identical to those used for immunohistochemistry. Sections were blocked using a solution of 5% milk/0.1 M Tris (pH 7.6) to prevent non-specific staining. Thereafter, sections were incubated overnight (4 °C) using combinations of primary antibodies diluted in 5% FBS/0.1 M Tris (pH 7.6) followed with subsequent incubation for 1 h at room temperature using secondary antibodies (diluted in 5% FBS/0.1 M Tris, pH 7.6) conjugated to Alexa 647, 594, or Alexa 488 (Invitrogen). Nonspecific fluorescence was quenched using 0.3% Sudan Black/70% ethanol. Sections were stained with 5 μg/mL 4′,6-diamindino-2-phenylindole. Sections were cover-slipped with Fluoromount-G (SouthernBiotech) and visualized using either an Olympus BX51 microscope mounted with a DP71 Olympus digital camera to capture images at 20x magnification or an Olympus IX81-DSU spinning disk confocal microscope equipped with a cooled FluoView II charge coupled device (CCD) digital monochrome camera.

### Western blot analysis

For biochemical characterization of fractionated human brain tissue, 20 μg of lysate from either the HS or SDS/urea fractions were loaded onto 15% polyacrylamide gels and resolved by SDS-PAGE, followed by electrophoretic transfer onto 0.2 μm pore size nitrocellulose membranes (Bio-Rad, Hercules, CA), in carbonate transfer buffer (10 mM NaHCO_3_, 3 mM Na_2_CO_3_, pH 9.9) [29]. For analysis of recombinant protein, 200 ng of WT or A53T human αsyn was analyzed. Membranes were blocked in 5% dry milk/Tris buffered saline (TBS) and incubated overnight at 4 °C with primary antibody diluted in block solution. After washing in TBS, membranes were incubated with goat anti-mouse secondary antibody conjugated to horseradish peroxidase (Jackson Immuno Research Labs, Westgrove, PA) diluted in 5% dry milk/TBS for 1 h at room temperature; immunocomplexes were detected using Western Lightning-Plus ECL reagents (PerkinElmer, Waltham, MA) followed by chemiluminescence imaging (PXi, Syngene, Frederick, MD).

### Quantitative analysis of immunohistochemical staining

Quantitation of staining positivity for total LRP was performed on cingulate and amygdala sections from 9 LBD cases. A modified form of a quantitation workflow was used, in which for each section three squares of a uniform size (0.25 mm^2^) were placed within the regions of densest pathology that were at least two box widths apart [75]. Adjacent sections were used, and so boxes were placed at nearly identical locations for sections stained with the different antibodies and retrieval conditions. The positivity within each box for each section was analyzed using the positive pixel count algorithm (Aperio) with intensity threshold values optimized for each antibody to maximize pathology detection and minimize background (Fig. 3). For a given antibody, the same threshold values were used for all amygdala and cingulate cortex sections as same day staining minimized variability in background. For each LBD case, positivity for each antibody with or without formic acid (FA) and for the cingulate versus amygdala were determined. Comparisons were made for positivity with different antibodies and between the cingulate cortex and amygdala using GraphPad Prism software with one-way analysis of variance (ANOVA) and post-hoc analysis using the Sidak multiple comparison test to individually compare each region and antibody.

## Results

### Pathological A53T αsyn recruits WT αsyn within pathologic inclusions in familial PD/LBD

**Fig. 1:**
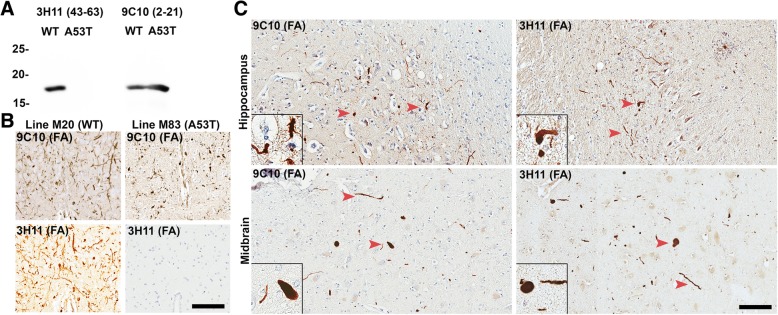
LRP comprised of WT αsyn detected by selective antibody 3H11. **a** Western blot of 200 ng recombinant WT or A53T human αsyn protein probed with antibody 9C10 (residues 2–21) or antibody 3H11 (residues 43–63); A53T αsyn does not react with antibody 3H11. **b** Immunohistochemical staining with antibody 3H11 or 9C10 in αsyn transgenic mice. Using antibody 3H11, αsyn aggregates are extensively detected within line M20 mice overexpressing WT human αsyn but not in line M83 mice overexpressing A53T human αsyn demonstrating the histochemical specificity of this antibody. Labeling with antibody 9C10 depicts αsyn pathology in both types of αsyn transgenic mice. Scale bar 50 μm. **c** Immunohistochemical staining of tissue from the midbrain and hippocampus of a familial case of PD/LBD due to a heterozygous A53T mutation in SNCA. Antibody 9C10 detects both WT and A53T αsyn and detects abundant pathology in both regions; Antibody 3H11 only detects WT αsyn but also labels many pathologic inclusions, indicating that WT αsyn is recruited to aggregate by the presence of the A53T αsyn mutation. Arrowheads indicate LRP. All the sections depicted were treated with FA. Scale bar 50 μm

Unique conformationally or post-translationally modified strain-like forms of pathologic αsyn that aggregate readily, and further seed inclusion pathology may differentiate rapidly progressive forms of synucleinopathy from more innocuous conditions such as AD/ALB and iLBD. In order to test the theory that an aggregation prone form of αsyn could induce endogenous physiologic αsyn to form pathologic inclusions, sections of hippocampus and midbrain were obtained from a Contursi kindred patient in which familial PD/LBD developed due to a heterozygous A53T mutation in the SNCA gene [27]. Antibody 3H11 which does not detect αsyn harboring the A53T mutation [92] was used to label pathologic aggregates comprised of physiologic WT αsyn and compared with LRP seen by antibody 9C10 which binds both WT and A53T αsyn (Fig. 1). Western blot analysis of recombinant WT and A53T αsyn confirmed the specificity of each antibody (Fig. 1a). Additionally, antibody 3H11 was further tested on transgenic mice containing pathologic aggregates comprised of A53T human αsyn (seeded line M83 [94]) or WT human αsyn (seeded line M20 [91]). As 3H11 does not detect mouse αsyn (that contains the A53T substitution) or A53T human αsyn, no pathology was revealed in the M83 mice using 3H11 whereas extensive LRP was labeled in the M20 mice (Fig. 1b). In both the hippocampus and midbrain of the Contursi kindred patient, 9C10 and 3H11 detected similar amounts of LRP indicating that WT αsyn was induced to misfold due to the presence of aggregates initially formed by A53T αsyn and recruited within pathological inclusions (Fig. 1c). These results demonstrate that WT αsyn can be induced to aggregate due to the presence of a more aggregation prone “strain” of αsyn, presumably in a prion-like fashion.

### The LBD and AD/ALB amygdala differ largely in burden of neuropil pathology

**Fig. 2:**
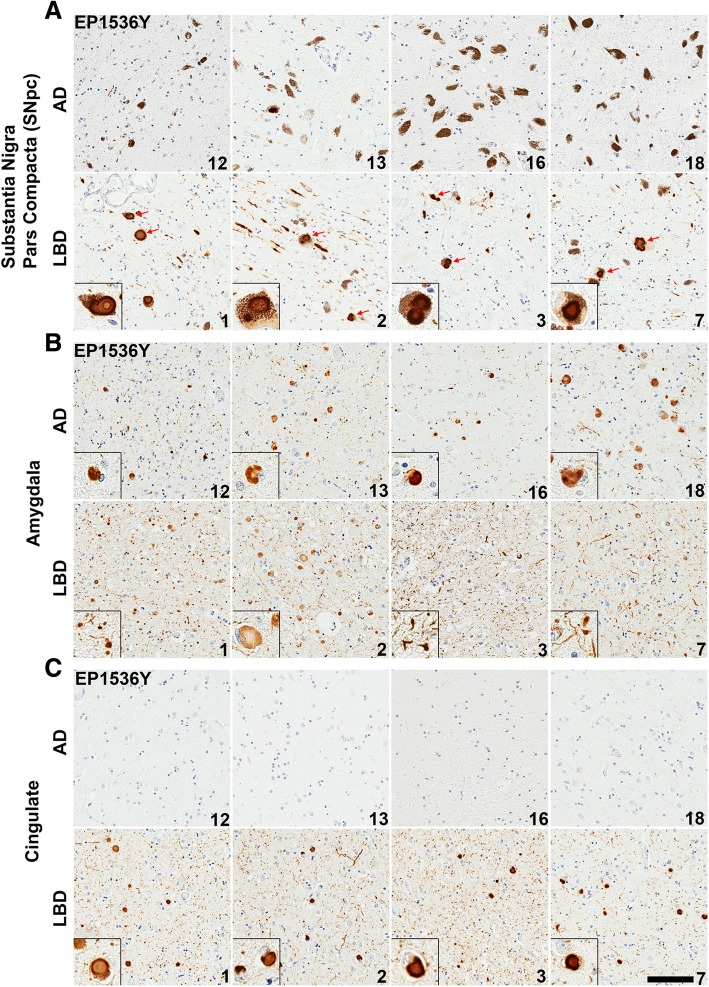
Comparison of LRP between LBD and AD/ALB. **a** Representative sections of the SNpc from 4 AD/ALB and 4 LBD cases stained with pSer129 αsyn antibody EP1536Y. Abundant LNs and LBs (red arrows) are seen within pigmented neurons in all LBD cases, however AD/ALB cases display only rare examples of thread like neurites. Insets display LBs in pigmented neurons. **b** Representative sections of the amygdala from AD/ALB versus LBD cases labeled with antibody EP1536Y. Cortical-type LBs are apparent in dense patches within amygdalas of either disease type, however abundant thread-like neurites are more apparent in LBD cases compared with AD/ALB. Insets highlight cortical LBs or neurites. **c** Representative sections of the cingulate cortex from AD/ALB compared with LBD using antibody EP1536Y. LRP is entirely absent in all examined AD/ALB cases, whereas LBD cases display extensive LBs in deeper cortical layers and dot like inclusions in more superficial layers. Case numbers are indicated in lower right corner. Scale bar 100 μm

In order to compare the immunohistochemical patterns of LRP found within the amygdalas of LBD and AD/ALB cases, 9 pathologically confirmed LBD cases were obtained along with 9 cases of AD/ALB where concurrent LRP is predominantly present within the amygdala upon inspection of 14 different regions ranging from the medulla to the frontal cortex (Tables 1 and 2). Sections from the cingulate cortex, amygdala, and substantia nigra pars compacta (SNpc) were obtained from each case as these 3 regions are heavily affected in LBD and provide confirmation that AD/ALB LRP is mainly localized to the amygdala. The overall burden of LRP within each region of each case was first studied using an antibody specific for pSer129 αsyn (EP1536Y) which is a marker of pathologic inclusion formation [2] (Fig. 2). The presence of pSer129 αsyn-positive inclusions was semi-quantitatively graded at 10x magnification on a four-tiered scale, with “-” representing non-reactivity, “+” mild, “++” moderate, and “+++” representing the strongest level of reactivity (Table 2). Immediately evident is the paucity of LRP in the cingulate cortex and SNpc of AD/ALB cases compared to parallel regions in LBD cases. In AD/ALB, 1/8 cases had rare thread like LNs in the SNpc with no LBs, and no cases of AD/ALB demonstrated any LRP in the cingulate cortex. Comparatively, all LBD cases had extensive LRP in the cingulate cortex and SNpc including LBs and pSer129 positive neurites; semi-quantitative grading for each region is shown (Fig. 2, Table 2). Furthermore, dopaminergic neuronal cell loss is not evident in the examined AD/ALB cases as abundant neuromelanin-laden neurons are present throughout the SNpc (10+ granulated neurons per 10x visual field) whereas overt reduction in dopaminergic neuronal cells was apparent in the LBD cases (1–5 granulated neurons per 10x visual field) (Fig. 2).

**Table 2:** Semi-quantitative grading of pSer129 αsyn pathology

pSer129 pathology			
	SNpc	Amyg.	Cing.
LBD			
Case 1	++	+++	+++
Case 2	+++	+++	+++
Case 3	++	+++	+++
Case 4	+++	++	+
Case 5	++	++	++
Case 6	++	+	++
Case 7	+++	+++	+++
Case 8	+++	+++	+++
Case 9	++	++	++
AD/ALB			
Case 15	–	−/+	–
Case 16	–	+	–
Case 17	−/+	++	–
Case 18	–	+++	–
Case 19	–	++	–
Case 20	–	++	–
Case 21	–	++	–
Case 22	–	+	–
Case 23	–	+++	–

Following immunohistochemical staining with antibody EP1536Y and no formic acid retrieval, semi-quantitative grading was assigned to each LBD and AD/ALB case for 3 separate regions (*SNpc* substantia nigra pars compacta, *cing.* cingulate cortex, *amyg.* amygdala). The presence of pSer129 positive inclusions was semi-quantitatively graded at 10x magnification on a multi-tiered scale, with “-” representing non-reactivity, “−/+” very rare, “+” mild, “++” moderate, and “+++” representing the strongest level of reactivity

Within the amygdala, varied LRP was present in the examined AD/ALB cases with 5/8 cases displaying 5–10 cortical LBs per visual field (10x magnification) in regions of densest pathology while 3/8 cases had less inclusions (Table 2). Comparatively, 5/8 LBD amygdala cases had 5–10 cortical LBs in a similar visual field of dense pathology and 3/8 cases had 10–20+ LBs. Although regions of the AD/ALB amygdalas displayed an LB load comparable to that of LBD amygdalas in the regions of densest pathology, abundant neuropil threads positive for pSer129 αsyn were ubiquitous within LBD cases but not to the same extent within AD/ALB cases (Fig. 2). The extensive neuritic pathology within amygdalas in LBD compared with amygdalas in AD/ALB may represent an important difference between these conditions, and aligns with the theory that large LBs serve to sequester misfolded αsyn while smaller neuritic aggregates contain the more active species in disease pathogenesis. Small synaptic aggregates of αsyn have been suggested as containing more toxic species compared with αsyn within compact LBs [14, 59, 89]. The nature of this amygdala neuropil pathology in LBD was further investigated with a panel of monoclonal antibodies raised against αsyn.

### The LBD amygdala harbors extensive and unique immunoreactive αsyn pathologies compared with other diseased brain regions and AD/ALB

**Fig. 3:**
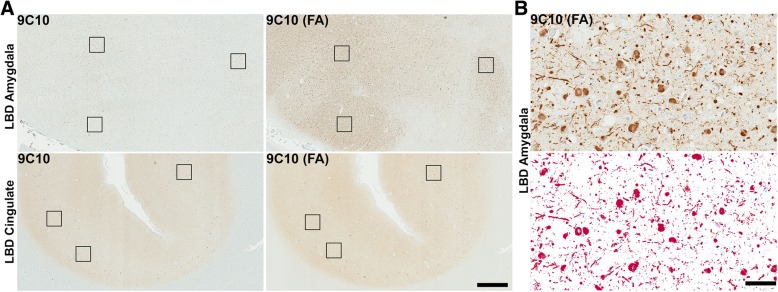
Significant brain regional difference in formic acid antigen retrieval of LRP. **a** Low magnification sections from the amygdala and cingulate cortex of an LBD case labeled with antibody 9C10 (residues 2–21 αsyn) without or with FA retrieval as indicated. FA retrieval had a major impact in increasing staining of αsyn aggregates in the amygdala compared with the cingulate cortex. Scale bar 1 mm. **b** A region of dense Lewy pathology within the amygdala was used to optimize threshold values for positive pixel count analysis. All antibodies were tested to ensure similar detection of pathology with minimal background as shown in bottom panel where red colored pixels are positive. Scale bar 50 μm

Sections from the cingulate cortex, midbrain, and amygdala from 9 LBD and 9 AD/ALB cases were utilized for comparison of the immunoreactive profiles of LRP amongst the differing brain regions using a panel of antibodies and different antigen retrieval conditions. This approach has been previously utilized to differentiate between distinct forms of LRP [22, 23, 26, 68, 80]. An N-terminal antibody 9C10 [22], a C-terminal antibody 94-3A10 [22], and a central αsyn antibody 3H11 [23] were chosen for morphologic assessment and quantitative analysis as distinct forms of pathologic αsyn may have differential exposure of various epitopes [8, 80] and/or post-translational modifications such as truncation, ubiquination, or phosphorylation that can result in immunoreactivity differences [2, 76, 93]. Antibody 5G4 that is preferential for oligomeric αsyn [56, 58] and antibody EP1536Y for pser129 αsyn were also utilized. Additionally, the reactivity of select antibodies was compared with and without FA retrieval as unique species of pathologic αsyn are known to be preferentially detected only in the presence of additional antigen retrieval techniques such as formic acid exposure and proteinase K digestion [5, 8, 23, 92, 105]. Staining positivity for each antibody with and without FA was quantitated with a modified Aperio based method [75] where threshold values were determined for each antibody to maximize pathology detection and minimize background (Fig. 3).

**Fig. 4:**
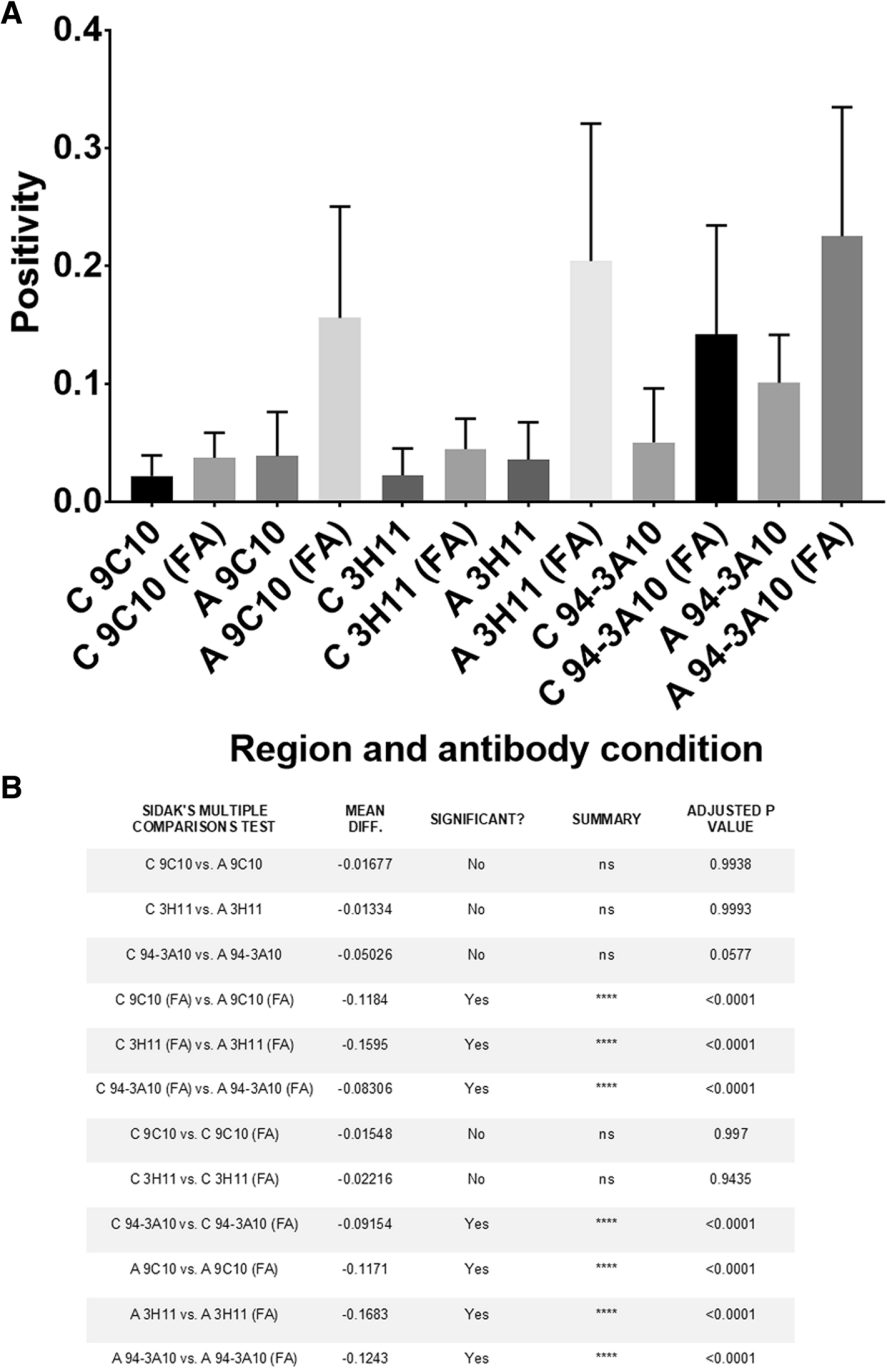
Quantitation of LRP in LBD brain regions across a panel of antibodies. **a** Three areas of dense pathology within the cingulate (C) or amygdala (A) of LBD (*N* = 9) cases stained with 3 different antibodies without or with (FA) retrieval were subject to positive pixel analysis; average positivity and error bars (std) are displayed for each region and antibody without or with FA. All cases were averaged for each region and antibody for comparison. Without FA retrieval, all antibodies detect similar amounts of LRP within the amygdala versus the cingulate cortex; with FA retrieval a large increase in labeled amygdala pathology is evident with all antibodies whereas a lesser increase in pathology is seen in the cingulate cortex and only with antibody 94-3A10. With FA retrieval, the average amygdala pathology burden is significantly greater than the cingulate cortex for all antibodies. **b** A statistical summary of positivity comparisons for data presented in A. The average positivity for each antibody between and within regions along with presence or absence of FA retrieval were tested for significant differences using one-way ANOVA and the Sidak post-hoc multiple comparisons test. Mean difference is the absolute difference in positivity values

**Fig. 5:**
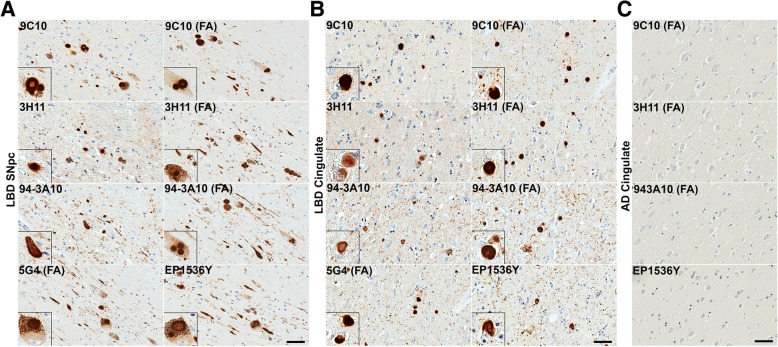
LRP in LBD SNpc and cingulate cortex is similarly detected across a panel of αsyn antibodies. **a** Representative sections of the SNpc from an LBD case labeled with 5 different αsyn antibodies as indicted in the top, left corners without or with FA as indicated. Insets display LBs in pigmented neurons. Within the SNpc, abundance of pathology is similar regardless of antibody used or antigen retrieval albeit with minor differences. Scale bar 50 μm. **b** Representative staining of the cingulate cortex from an LBD case labeled with 5 different αsyn antibodies without or with FA as indicated. Insets display cortical LBs. Within the cingulate, pathology is similar for most antibody and antigen retrieval conditions. αsyn-positive neurites are slightly more apparent with FA retrieval or C-terminal antibodies, particularly 94-3A10 with FA which is reflected in the quantitative positivity analysis. Scale bar 50 μm. **c** Sections from the cingulate of an AD/ALB case with no LRP were stained with 4 αsyn antibodies without or with FA, as indicated. Little to no positive staining was detected in the AD/ALB cingulate cortex with any of these antibodies. Scale bar 50 μm

**Fig. 6:**
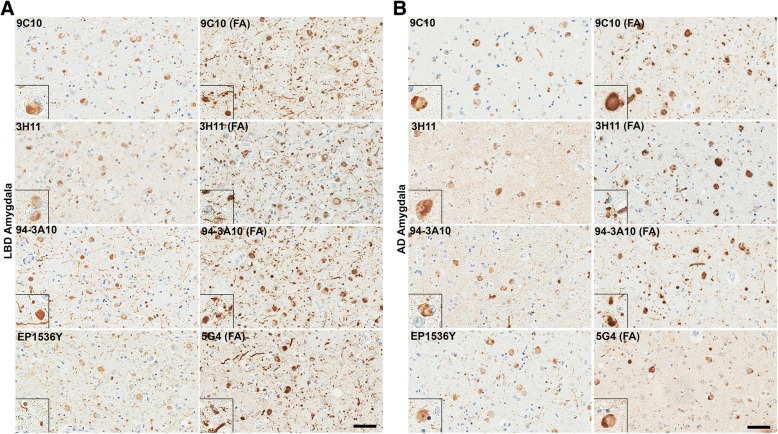
Comparison of LRP in the amygdala of LBD versus AD/ALB across a panel of αsyn antibodies. **a** Representative sections of dense amygdala pathology from a LBD case labeled with 5 different αsyn antibodies without or with FA retrieval, as indicated. Insets display cortical LBs and αsyn-positive neurites. In the LBD amygdala, extensive neuritic pathology is detected for multiple antibodies when FA retrieval was used. The robust increase in apparent Lewy pathology with FA treatment is mainly seen in neuritic and possible glial processes; the amount of cortical LBs per visual field remain the same regardless of antibody or FA treatment. This immunohistochemical staining profile contrasts with the only modest increase in detected pathology seen in the LBD SNpc and cingulate cortex. Scale bar 50 μm. **b** Stained sections of amygdala pathology from an AD/ALB case labeled with 5 different αsyn antibodies without or with FA retrieval, as indicated. Insets display cortical LBs and αsyn-positive neurites. In the AD/ALB amygdala, neuritic pathology is modestly enhanced for multiple antibodies when FA retrieval is used; however, the density of neuropil αsyn staining pathology in LBD is not apparent in AD/ALB. Scale bar 50 μm

Quantification and morphologic assessment of staining with each αsyn antibody and no FA retrieval was performed on cingulate cortex and amygdala sections for each LBD case (Figs. 4, 5 and 6, Additional file 1: Figure S1). For all antibodies used, there were no significant differences in positivity between the cingulate cortex and amygdala without FA; averaging the 3 antibodies used the positivity in LBD cingulate cortices was 3.2 ± 3.3% and in LBD amygdalas it was 5.7 ± 4.6%. Semi-quantitative grading using pSer129 antibody EP1536Y without FA retrieval also suggested no difference in the αsyn pathology burden between the cingulate cortex and amygdala in examined LBD cases (Table 2). When FA was used for antigen retrieval, detection of neuropil pathology in the amygdala was greatly increased with all antibodies which is reflected in the highly significant increase in positivity of staining for 9C10, 94-3A10, and 3H11 (Figs. 3, 4 and 6). On average, the largest increase in detection with FA was using the central αsyn antibody 3H11 which went from a positivity in LBD amygdalas of 3.6 ± 3.1% to 20.4 ± 11.4%, a roughly 6-fold increase in detected pathology. Including all antibodies, positivity in LBD amygdalas was 19.5 ± 10.5% with FA compared to 5.7 ± 4.6% without. The LBD cingulate cortex sections did not display this substantial increase in detected pathology when FA was included, and no significant difference in quantification of positivity was present with tested antibodies with or without FA except for antibody 94-3A10 which had a more modest increase in detected neuropil pathology (Figs. 3, 4 and 5). Including all antibodies, positivity in LBD cingulate cortices was 7.3 ± 7.0% with FA compared to 3.2 ± 3.3% without. This pattern of immunoreactivity suggests that this additional αsyn pathology within the LBD amygdalas may be distinct from aggregates elsewhere in the brain, as may be the case with glial αsyn inclusions detected with FA in multiple systems atrophy [23].

**Fig. 7:**
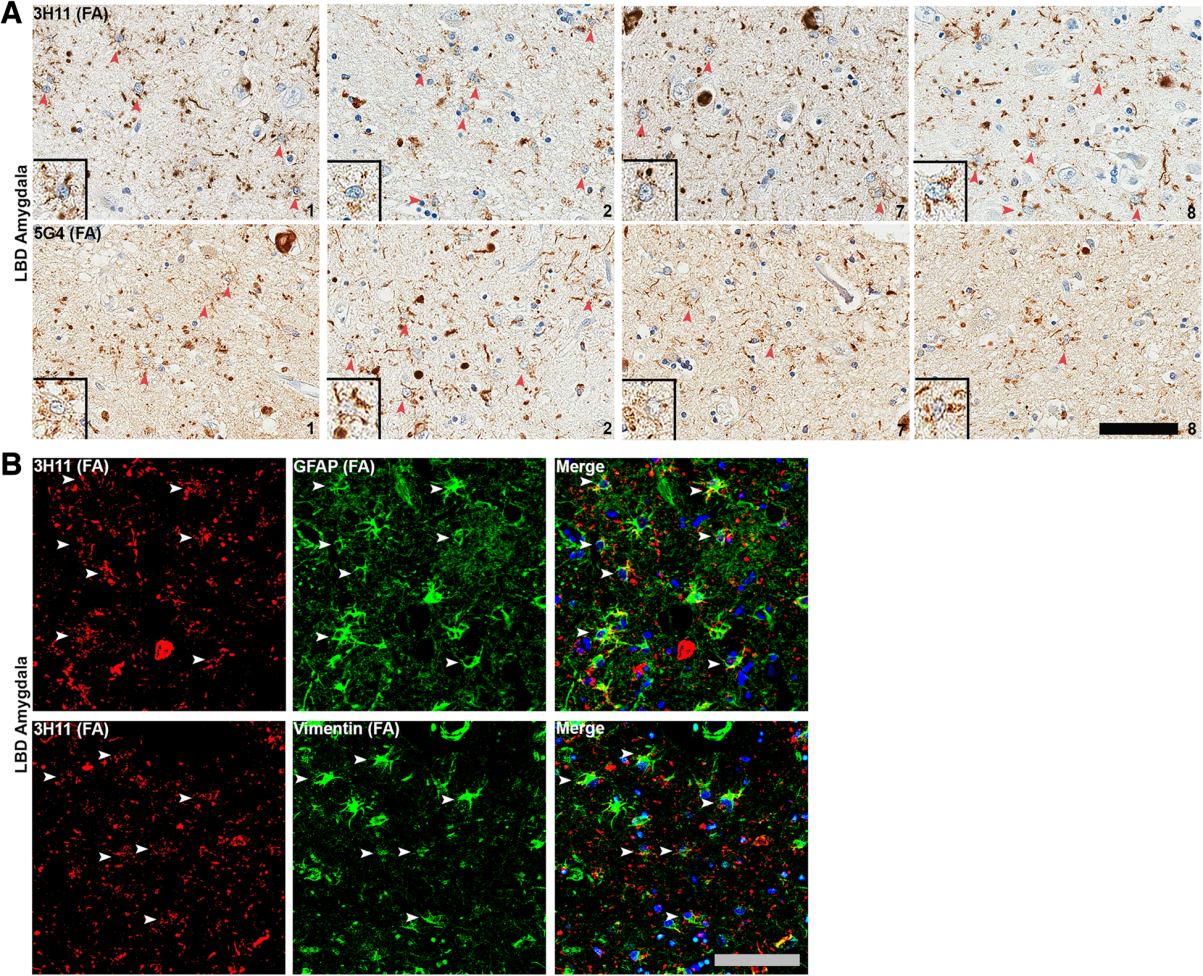
αsyn astrocytic pathology is common in LBD amygdala. **a** Amygdala sections from 4 different LBD cases (1, 2, 7 and 8) were labeled with central αsyn antibodies either 3H11 or 5G4 along with FA retrieval. Typically, more than 5 cells with astrocytic morphology (depicted with arrowheads) stained with these antibodies was readily observed per field; insets show examples for each case and each antibody. Although astrocytic αsyn inclusions may be present in other brain regions, they are only this densely abundant within the MTL, particularly the amygdala. Cases are indicated in lower right corner. Scale bar 50 μm. **b** Double labeling immunofluorescent analysis of LBD amygdala sections using central αsyn antibody 3H11 in conjunction with astrocytic markers GFAP or vimentin. Astrocytic αsyn often appears as vesicular granules outlining 2–3 astrocytic processes as opposed to the more uniform αsyn staining seen within nearby cortical LBs, suggesting a differing subcellular co-localization or form of aggregates. Scale bar 30 μm

**Fig. 8:**
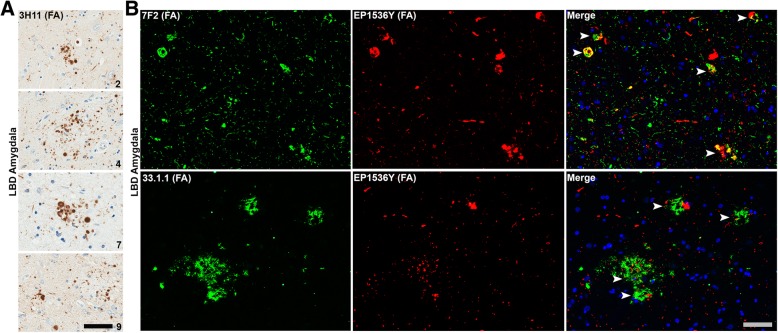
αsyn aggregates within the LBD amygdala often co-localizes with Alzheimer’s disease-type inclusion pathology. **a** Amygdala sections from 4 different LBD cases (2, 4, 7 and 9) stained with central αsyn antibody 3H11 along with FA retrieval reveal abundant αsyn dystrophic neurites within Aβ senile plaques. These αsyn-positive dystrophic neurites within Aβ plaques were common within the LBD amygdalas but rare in the AD/ALB amygdalas examined. Cases are indicated in lower right corner. Scale bar 50 μm. **b** Double labeled immunofluorescence microscopy of LBD amygdala sections from 2 different LBD cases using rabbit monoclonal anti-pSer129 αsyn antibody EP1536Y and mouse monoclonal anti-pThr205 tau antibody 7F2 or mouse monoclonal anti-Aβ antibody 33.1.1. Co-localization of tau and αsyn aggregates was common in the LBD amygdala both in neuronal cell bodies and processes. Scale bar 50 μm

Panels of immunohistochemical sections are shown to compare the distinct morphologies and scale of additional LRP detected within the LBD amygdala using FA treatment compared to without (Figs. 3 and 6). Within multiple LBD amygdalas, a dramatic increase in detected αsyn pathology is appreciated even at low magnification as thousands of neuropil aggregates (per 2x visual field) become apparent with FA compared to without; this increase in not seen in the cingulate cortex within the same case (Fig. 3). Within examined LBD amygdalas at high magnification, without FA all antibodies demonstrate cortical LBs and αsyn-positive neuropil threads similarly to the cingulate cortex (Figs. 5 and 6). With FA retrieval for all antibodies, the number of detected cortical LBs (per 10x visual field) remains largely unchanged in LBD amygdalas; the major increase in stained αsyn pathology is due to abundant αsyn-positive neuropil threads including processes with astrocytic morphologies, and dystrophic neurites within senile plaques (Figs. 6, 7 and 8). For the LBD cingulate cortex sections, at high magnification the addition of FA treatment does not reveal as abundant unique pathologies as is the case with the amygdala (Fig. 5); only a minor increase in detection of αsyn-positive neuropil threads is seen (particularly with C-terminal antibody 94-3A10) and the astrocytic inclusions and neuritic plaque findings are not apparent. In the SNpc, brainstem LBs and engorged LNs were present in all LBD cases detected with all antibodies (Fig. 5). Qualitatively, no additional burden or type of αsyn pathology was detected in the SNpc of LBD cases with any antibodies when FA was used versus without; similarly to the cingulate cortex, only a slight enhancement of neuropil threadlike pathology may be seen particularly with antibody 94-3A10 (Fig. 5). These findings reinforce that abundant and unique forms of pathologic αsyn are present within the amygdala and none of the other examined brain regions in LBD.

As the previously examined AD/ALB cases upon semi-quantitative grading with pSer129 αsyn antibody EP1536Y displayed less baseline neuropil pathology compared with LBD cases in the amygdala (Table 2), FA antigen retrieval was performed on these sections as well. Qualitatively, with FA retrieval there was no dramatic enhancement of neuropil pathology in the AD/ALB amygdalas compared with the LBD amygdalas (Fig. 6). Similarly to LBD, with all antibodies tested the number of cortical LBs per 10x field remained constant with or without FA; upon inspection a slight qualitative increase in neuropil threads may be appreciated in the AD/ALB amygdalas however it is not the multifold quantifiable increase as is seen in LBD. Additionally, αsyn astrocytic inclusions and αsyn-positive dystrophic neurites within senile plaque findings in AD/ALB are fewer compared to LBD. The midbrain and cingulate cortex sections from the AD/ALB cases still had no apparent αsyn pathology using the tested panel of antibodies (9C10, 3H11, 94-3A10, 5G4, EP1536Y) with or without FA. These results suggest that the extensive neuropil pathology present within the LBD amygdala is unique not only regionally within LBD, but also distinguishes between LBD and AD/ALB.

### Astrocytic αsyn is distinctively common within the LBD amygdala

The presence of αsyn-positive astrocytes in synucleinopathies has been previously reported, and their presence is thought to be particularly common within MTL structures [92]. Additionally, these astrocytes are reported to contain αsyn predominantly reactive to central αsyn antibodies in conjunction with FA retrieval and not N or C-terminal antibodies [92]. Herein, the presence of these αsyn-positive astrocytes was compared between brain regions within LBD and for LBD versus AD/ALB to determine whether the astrocytic burden of αsyn contributes to the unique enhancement of pathology within the LBD amygdala upon FA antigen retrieval. Astrocytic αsyn pathology identified based on morphology was detected with central αsyn antibody 3H11 following FA antigen retrieval (Fig. 7). Findings were confirmed using a separate central αsyn antibody previously reported to detect αsyn-positive astrocytes in disease, 5G4 [56, 58]. For each LBD and AD/ALB case, astrocytic αsyn pathology was semi-quantitatively graded at 10x magnification on a four-tiered scale, with “-” representing non-reactivity, “+” mild, “++” moderate, and “+++” representing the strongest level of reactivity (Table 3, Fig. 7).

**Table 3:** Semi-quantitative grading of astrocytic αsyn pathology

Astrocyte pathology			
	SNpc	Amyg.	Cing.
LBD			
Case 1	–	+++	–
Case 2	–	+++	–
Case 3	–	++	–
Case 4	–	++	–
Case 5	–	++	–
Case 6	–	+	–
Case 7	–	+++	–
Case 8	–	++	–
Case 9	–	++	–
AD/ALB			
Case 15	–	–	–
Case 16	–	–	–
Case 17	–	+	–
Case 18	–	+	–
Case 19	–	–	–
Case 20	–	–	–
Case 21	–	–	–
Case 22	–	–	–
Case 23	–	+	–

Following immunohistochemical staining with central αsyn antibody 3H11 and formic acid retrieval, semi-quantitative grading was assigned to each LBD and AD/ALB case for 3 separate regions (*SNpc* substantia nigra pars compacta, *cing.* cingulate cortex, *amyg.* amygdala). The presence of astrocytic inclusions based on morphology was semi-quantitatively graded at 10x magnification on a multi-tiered scale, with “-” representing non-reactivity, “+” mild, “++” moderate, and “+++” representing the strongest level of reactivity

Within the LBD amygdala sections stained with 3H11, in regions of dense pathology there were frequently 5+ cells (per 10x visual field) with an astrocytic morphology positive for αsyn staining (Fig. 7). Overall, 3/9 LBD cases had frequent astrocytic αsyn pathology, 5/9 LBD cases had intermediate, and 1/9 had rare astrocytic αsyn pathology (Table 3). Examples of αsyn-positive inclusions consistent with astrocytic morphology are shown for 4 different LBD cases and stained with 2 central αsyn antibodies (Fig. 7). Consistent with previous findings, αsyn-positive astrocytes were only readily apparent when the combination of a central αsyn antibody and FA retrieval were used [92]; N and C-terminal antibodies 9C10 and 94-3A10 did not obviously label inclusions with an astrocytic morphology in the LBD cases used. Although astrocytic αsyn inclusions have been noted in other studies to be rarely present in the SNpc and other brain regions [92], upon inspection they were not nearly as apparent within these regions compared to the amygdala. Within AD/ALB amygdala sections, 3/8 cases displayed rare examples of αsyn inclusions consistent with astrocytic morphology when using a central αsyn antibody with FA retrieval (Table 3, Fig. 6). These results suggest that astrocytic αsyn pathology is distinctively common within the amygdala in LBD compared with other brain regions and diseases, and additionally the form of αsyn present within these cells may be further unique in the apparent reactivity only with central αsyn antibodies possibly due to N and C-terminal truncation or other structural modifications to the protein.

Using double labeling immunofluorescence for αsyn with the central αsyn 3H11 antibody alongside two different astrocytic markers, GFAP or vimentin, the nature of αsyn within astrocytes in LBD was further investigated (Fig. 7). Within the LBD amygdala, frequent co-localization of αsyn was seen within astrocytic processes in two separate cases. In particular, αsyn labeling was “granular” in astrocytes compared with the more evenly labeled nearby neurites and cortical LBs (Fig. 7). This granular pattern of staining suggests localization of αsyn aggregates to vesicular structures such as lysosomes. These results further suggest that unique αsyn aggregates are present within astrocytes, and these are largely localized to the amygdala in LBD compared with other brain regions and diseases with αsyn pathology.

### The amygdala in LBD contains frequent αsyn aggregates co-localized with other AD- associated amyloidogenic proteins

The amygdala has early and heavy involvement in a number of neurodegenerative diseases including AD with pathologic tau neurofibrillary tangles and Aβ senile plaques [73]. MTL regions such as the hippocampus and particularly the amygdala have previously been noted to develop co-pathologies that are not common in other brain regions; for example, tau tangles and cortical LBs have been labeled within the same neuron in these areas [27, 46, 88, 113]. The presence of these co-pathologies in the LBD amygdala was studied herein and qualitatively compared to other LBD brain regions and to AD/ALB. Indeed, within the LBD amygdala there are abundant examples of αsyn immunoreactive dystrophic neurites within senile plaques that were present in all LBD cases examined; examples from four cases are shown (Fig. 8). This finding was confirmed by double labeling analysis using an Aβ antibody 33.1.1 and pSer129 αsyn antibody (Fig. 8). These αsyn-positive senile plaques in LBD are qualitatively far more common within the amygdala compared to the cingulate cortex or SNpc; 2–3 αsyn-positive senile plaques are apparent per 10x visual field within regions of dense pathology in the amygdala but are rarely, if ever seen in the cingulate cortex and the SNpc. Senile plaques positive for αsyn were present more sparsely within AD/ALB. Additionally, as with most other αsyn pathologies in the LBD amygdala these αsyn-positive dystrophic neurites were best appreciated following FA antigen retrieval. When double labeling for both hyper-phosphorylated tau (pThr205) and pSer129 αsyn, aggregates of αsyn and tau were commonly co-localized (Fig. 8). Although this tau and αsyn co-localization has been described for neurofibrillary tangles and cortical LBs, many dystrophic neurites within neuritic plaques contained both αsyn and tau upon inspection of double labeled LBD amygdala sections (Fig. 8). Co-localization of pathologic tau and αsyn was mainly found within the LBD amygdala and not commonly in the LBD cingulate and SNpc. Co-pathologies with tau and Aβ may contribute to unique αsyn alterations present within the amygdala in LBD.

### Detergent insoluble αsyn within the medial temporal lobe in LBD is extensively modified

**Fig. 9:**
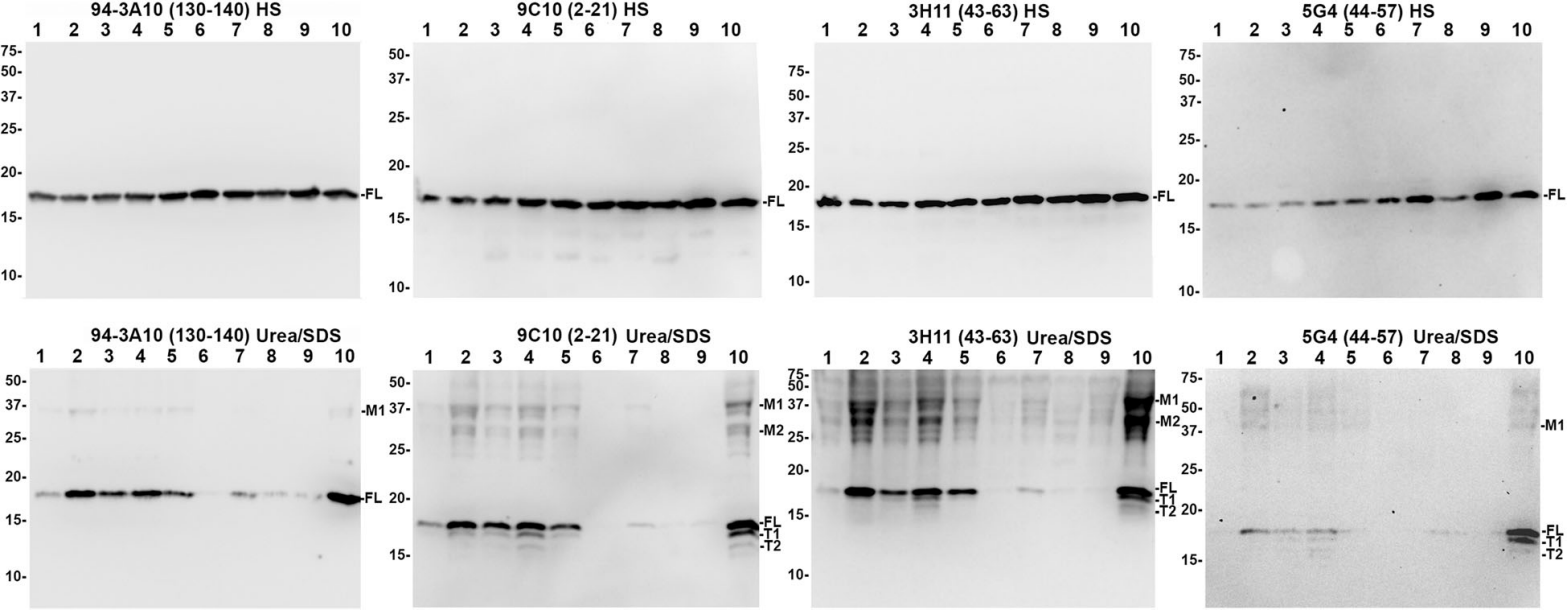
Immunoblotting comparison of αsyn species in LBD and AD/ALB amygdala. High salt (HS) and SDS/urea fractions were obtained from the MTL of 5 LBD cases (lanes 1–5), 2 cases of AD/ALB (lanes 6–7), 2 non-synucleinopathy controls (lanes 8–9), and for one LBD case the amygdala was specifically isolated (lane 10). 20 μg of lysate for each case and fraction were subject to western blot analysis using a panel of 4 antibodies which are indicated. In the HS fraction, all antibodies predominantly revealed full-length (FL) αsyn in all cases. In the SDS/Urea fractions, monomeric FL αsyn is present in high amounts for 4/5 LBD cases (lanes 2, 3, 4, 5, and 10); 1 LBD and 1 AD/ALB case (lanes 1 and 7, respectively) have an intermediate amount of FL αsyn in this fraction and 1 AD/ALB case along with 2 controls (lanes 6, 8, and 9) have very little FL αsyn in this fraction. For the LBD cases, 2 prominent truncation bands are present for all antibodies except for C-terminal antibody 94-3A10 suggesting these are carboxy-truncated forms of αsyn (T1 and T2) in the SDS/urea fractions from the MTL in LBD but not controls or AD/ALB; the truncation bands are strongest in the LBD amygdala. Additional higher molecular mass bands are prominent in LBD cases (M1 and M2); these bands are less visible in AD/ALB or controls. The relative mobilities of molecular mass protein markers are identified on the left of the blots

**Fig. 10:**
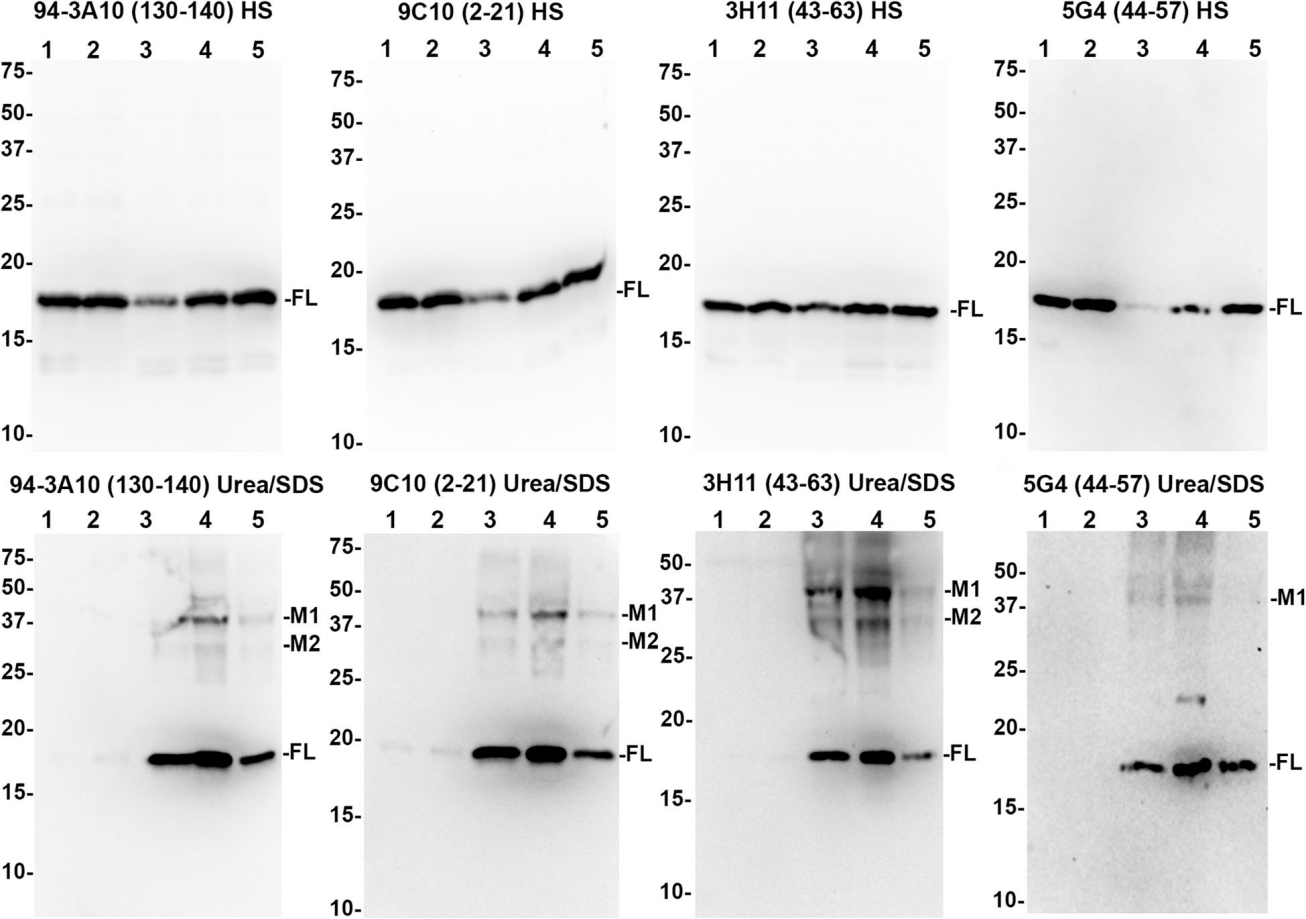
Immunoblotting comparison of αsyn species in LBD cingulate cortex. High salt (HS) and SDS/urea fractions were obtained from the cingulate cortex of 2 control cases (lanes 1–2) and 3 LBD cases (lanes 3–5). 20 μg of lysate for each case and fraction were subject to western blot analysis using a panel of 4 antibodies, as indicated. In the HS fraction, all antibodies demonstrate similar amounts of monomeric FL αsyn except for one of the LBD cases which had a diminished level. In the SDS/Urea fractions, monomeric FL αsyn is present in high amounts for all LBD cases while the 2 controls have almost no αsyn in this fraction. Although higher molecular mass M1 and M2 bands are present in the cingulate SDS/urea fraction, truncation bands T1 and T2 are not as apparent which represents a biochemical difference between αsyn in the amygdala and MTL compared with cingulate cortex. The relative mobilities of molecular mass protein markers are identified on the left of the blots

In order to better understand the unique histological immunoreactivity of αsyn within the amygdala in LBD, sequential biochemical fractionation of frozen brain tissue followed by western blot analysis with N-terminal (9C10), C-terminal (94-3A10), and central αsyn (3H11 and 5G4) antibodies was performed. The MTL was retrieved from multiple cases including 5 LBD, 2 AD/ALB, and 2 controls without evidence of synucleinopathy (Table 1). During tissue retrieval, for one case of LBD the amygdala was able to be confidently identified within the MTL and further isolated. For comparison, tissue from the cingulate cortex was also retrieved from 3 cases of LBD and 2 controls without evidence of synucleinopathy (Table 1). Following biochemical fractionation, the HS fractions harboring soluble αsyn and the SDS/urea fractions containing the most insoluble, aggregated αsyn species were compared via western blotting analysis for all aforementioned cases and brain regions (Figs. 9 and 10).

Within the HS fractions from the MTL, a single major band is detected for all cases using all 4 antibodies at ~17kda which is monomeric full-length (FL) αsyn (Fig. 9). Although αsyn has a predicted molecular mass of 14.4 kda, it is known to migrate at a larger apparent molecular mass [108]. For LBD lysates, a qualitatively small decrease in amount of HS soluble αsyn is evident compared with the controls and AD/ALB; this is most apparent with the 5G4 antibody. Additionally, minor truncation products are visible particularly with N-terminal antibody 9C10 (Fig. 9). The HS fractions from cingulate cortex lysates for LBD and control cases similarly display a single major band which is monomeric FL αsyn; minor amounts of truncated αsyn are also present in these lysates (Fig. 10). The HS fractions did not reveal any significant differences in the biochemical composition of αsyn between the different brain regions and cases.

Within the SDS/urea fractions from the MTL, 4–5 major bands are present when probed using the various αsyn antibodies. With C-terminal antibody 94-3A10, monomeric FL αsyn is detected in the SDS/urea fraction prominently for 4/5 cases of LBD, with the greatest amount present in the isolated LBD amygdala. For the 2 AD/ALB cases, only 1 had a modest amount of SDS/urea monomeric αsyn which was detected with all 4 antibodies. The 2 control cases contained minimal amounts of SDS/urea monomeric αsyn across all antibodies. In addition to monomeric FL αsyn, N-terminal antibody 9C10 and central αsyn antibodies 3H11 and 5G4 were able to detect 2–3 apparently truncated forms of αsyn within LBD MTL and amygdala fractions (Fig. 9). These truncated species appear to be carboxy-truncated, as they are not detected with the C-terminal antibody 94-3A10. Within the amygdala in particular, the heavier major truncation band (T1) comprises a large portion of all αsyn within the fraction; indeed, antibody 5G4 which is preferential for oligomerized αsyn strongly detects the T1 and FL bands extensively within the amygdala LBD fraction (Fig. 9). Both the T1 band and lighter major truncation band (T2) are present within 4/5 LBD MTL fractions, whereas they are not seen in the AD/ALB or control fractions with any of the 3 antibodies that can detect carboxy-truncated αsyn (9C10, 3H11, and 5G4). Truncated αsyn is not readily apparent within the SDS/urea LBD cingulate cortex fractions, whereas the T1 and T2 truncation bands are abundant in the LBD MTL and especially the amygdala (Figs. 9 and 10). The presence of carboxy-truncated αsyn may play an important role in differing pathologies between the amygdala and other brain regions as carboxy-truncation alters important biochemical aspects of αsyn aggregation [93].

The SDS/urea fractions from the MTL also reveal higher molecular mass αsyn species abundant within the LBD fractions but qualitatively less within control and AD/ALB cases (Fig. 9). Major heavy bands M1 and M2 are approximately ~ 28 kda and ~ 36 kda respectively which could represent ubiquinated forms of αsyn [87]. Alternatively, these bands could be oligomeric αsyn with varying amounts of additional post-translational modifications which is stated to be the preferred target for antibody 5G4 [56]. C-terminal antibody 94-3A10 does not readily detect these high molecular weight bands, whereas N-terminal antibody 9C10 and especially central αsyn antibody 3H11 may detect more αsyn within these bands than in the monomeric αsyn band (Fig. 9). This pattern of band reactivity for M1 and M2 may suggest that the αsyn species contained are modified in multiple ways such as being both N and C-truncated. Furthermore, with antibodies 9C10 and 3H11 there are additional minor amounts of heavy protein bands present in the MTL but not the cingulate cortices of the control cases (Figs. 9 and 10). In particular, αsyn truncation appears most abundant in the LBD amygdala fraction compared with other fractions. These results suggest that post-translational modifications to αsyn within the amygdala may underlie the unique immunohistochemical staining profile for the LBD amygdala.

## Discussion

The amygdala may be uniquely prone to pathologic developments in a number of neurodegenerative diseases [73]. Exemplifying this, neuritic senile plaques containing Aβ, tau, and αsyn demonstrate the propensity for pathologic aggregation to occur within the amygdala (Fig. 8). Other studies have noticed unique relationships between the amygdala and αsyn; for example, injections of pre-formed αsyn fibrils into various mouse models at differing locations invariably lead to amygdala pathology [1, 7, 13, 65–67, 78, 91, 94], and the amygdala uniquely displays immense upregulation of αsyn in relation to alcohol and opiate abstinence following addiction [114]. The studies herein demonstrate that pathologic αsyn within the amygdala in LBD is unique both in its immunohistochemical properties and immunoblotting profile which may differentiate aggregated forms of αsyn in LBD from the more innocuous AD/ALB. In particular, the presence of aggregation prone carboxy-truncated forms of αsyn within the amygdala may play an initiating role in the disease process as these species are able to misfold and induce endogenous FL αsyn to also aggregate [93]. Our investigations show using diseased human tissue that endogenous WT αsyn is induced to form pathologic inclusions when driven by the more aggregation prone A53T αsyn [15]; we postulate that a more pernicious form of αsyn (due to truncation or other structural modifications) may play a similar role as A53T αsyn in sporadic synucleinopathies. The early involvement of the amygdala in LBD along with sporadic LRP often being entirely restricted to the amygdala indicates that this brain region has the capacity to initiate αsyn misfolding and suggests that aggregation prone forms of αsyn are present and may originate in this region. It is difficult to identify and isolate the more deleterious forms of αsyn from diseased human brain tissue, but comparative immunohistochemical and immunoblotting αsyn profiles amongst various LBD brain regions and between LBD versus AD/ALB amygdalas indicate that there is in fact unique pathological forms of αsyn ubiquitous within the LBD amygdala that differs from other brain regions and disease entities.

The pervasive amount of αsyn neuropil pathology present within the amygdala compared to other brain regions has symptomatic relevance as a number of studies have demonstrated the importance of these smaller aggregates compared with larger LB type inclusions [14, 57, 59, 89]. It has been noted that neuropil pathology, presumably a large portion of which are small pre-synaptic aggregates, correlate more extensively with symptomatic severity compared to LBs [14, 57, 59, 89]. Conversely, it has been observed that the abundance of LBs restricted to the amygdala in AD/ALB have little to no impact on prognosis [74, 86, 102]; our study suggests that the relative sparsity of neuropil pathology in AD/ALB even following FA retrieval compared with LBD might be a key difference between these conditions. Other studies have noted the prevalence of large cortical LBs compared with neuropil pathology in AD/ALB as well, strengthening the findings herein [45, 102]. It is conceivable that structurally distinct forms of αsyn may have increased pathologic potency due to resistance to degradation or sequestration, and/or from more exposed amyloidogenic regions allowing faster prion-like templating of endogenous physiologic αsyn [101]. Differential resistance to proteases is commonly used to distinguish αsyn strains [35, 80], and structurally distinct mutant forms of αsyn are noted to resist lysosomal degradation [17]. Indeed, a pathologic correlate may exist amongst PD and LBD subtypes with more rapid onset and regionally diffuse pathology as it is suggested that rate of aggregate formation is increased likely due to a unique αsyn species [19, 36]. It would be expected that small pre-synaptic aggregates would predominate if a more pathologically potent αsyn was present, as monomeric αsyn is most abundant within the pre-synapse which would be induced to aggregate at that location and subsequent aggregates if resistant to clearance would stay localized to this subcellular location as opposed to sequestration through an aggresome-related process in a cytoplasmic LB which is one theory for authentic LB formation [105]. Some proposed models of αsyn mediated toxicity and prion-like spread center on dysfunction with axonal and dendritic processes where these smaller aggregates are presumably located and not the cell soma where large LBs are typically found [52, 104, 110]. Supporting the importance of neuropil pathology, exposure of neurons to αsyn fibrils is known to induce axonal deficits before any overt cell death [31]. Additionally, we and others have shown that a portion of αsyn-positive neuropil inclusion pathology is within astrocytes which are themselves adversely affected by αsyn aggregates and may have their own role in neurodegeneration and prion-like spread [12, 92]. Indeed, when observing past reports of pathology in Contursi kindred patients who have A53T human αsyn, it has been repeatedly noted that αsyn-positive neuropil threads and dot like inclusions are abundant while true LBs are more sparse [27, 55, 111]. A pathologic presentation of abundant αsyn neuropil aggregates with fewer LBs may be associated with a more aggressive disease course; in light of our findings and prior observations, it is likely that a distinct form of misfolded αsyn may favor the formation of neuropil pathologies which may be the pathologic determinant of disease progression.

**Fig. 11:**
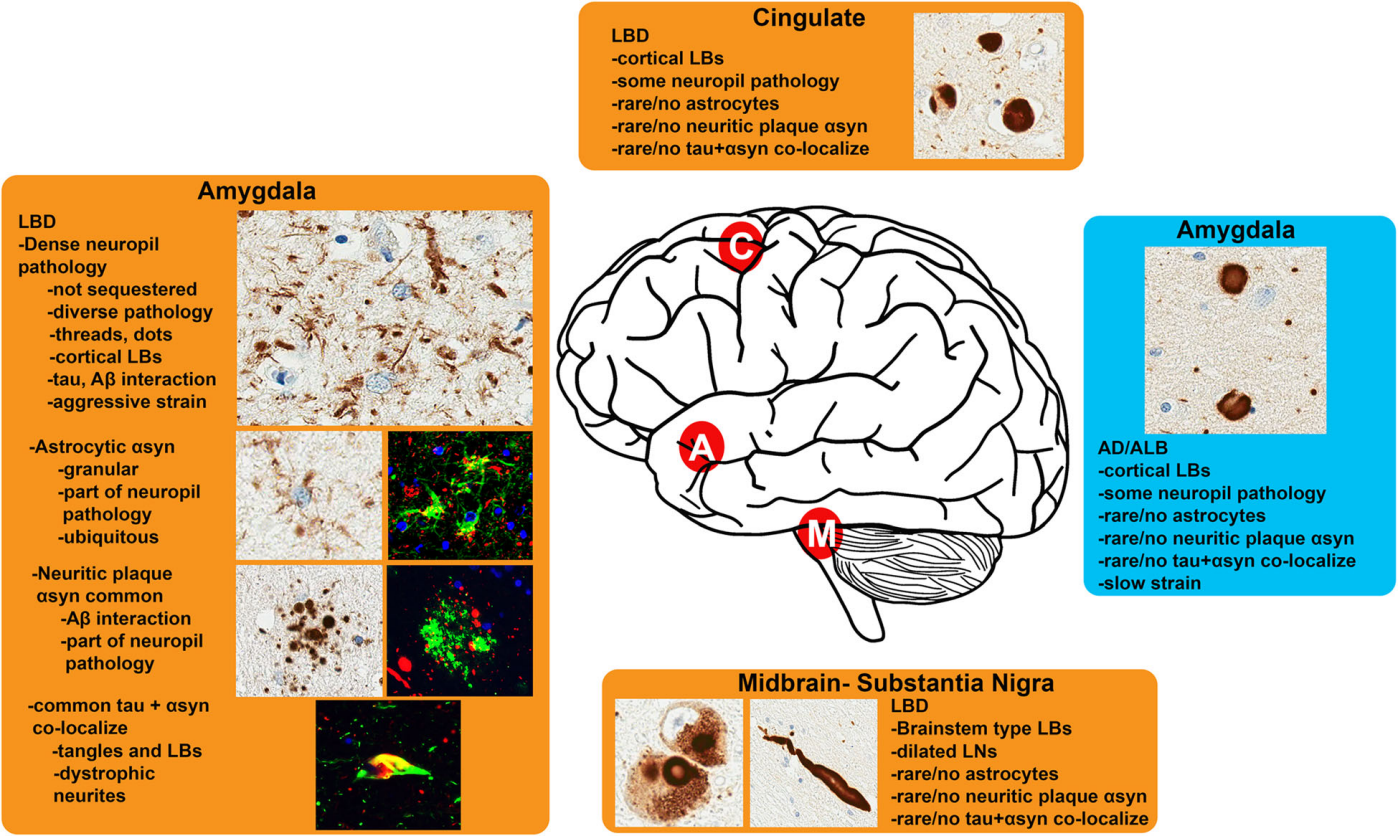
Diagram of pathologic determinants associated with further progression of αsyn pathology. The diversity of αsyn pathologies across different brain regions in LBD is shown in orange while the more limited αsyn pathology in AD/ALB is in blue (C = cingulate cortex, A = amygdala, M = midbrain-substantia nigra pars compacta). In LBD, diverse αsyn pathologies are most abundant within the amygdala where common co-localization with tau and Aβ are seen along with dense αsyn-positive neuropil aggregates including in glial processes that may represent a less easily sequestered strain of αsyn. In AD/ALB, αsyn pathology is largely limited to large LB type inclusions that may represented a more sequestered and less pathogenic strain of αsyn

Why αsyn misfolds into pathological aggregates remains largely unclear, and what underlies the diversity in the alleged strains of aggregated αsyn is even hazier. However, one major aspect of the amygdala that likely plays a role in influencing strain diversity is the concurrence of tau and Aβ aggregates which may all interact with monomeric or misfolded αsyn to induce structural alterations from which deleterious outcomes may result [33, 50, 55, 73, 77]. Indeed, amongst PD cases with more rapid development of dementia there is greater pathologic burdens of tau or Aβ [36, 52]; similar findings exist for LBD where concurrent AD pathology burden is highly predictive of symptomatic decline [43, 44, 52]. The αsyn-positive senile plaques and co-localized tau and αsyn aggregates shown in this paper are highly prevalent within the MTL and particularly the amygdala [18, 46, 47, 88, 109]. In AD, interactions of Aβ with tau have been postulated to occur within the amygdala and MTL, where induced conformational alterations in tau may refine the misfolded protein’s pathologic properties [73]. A similar phenomenon may occur in LBD, where virtually all cases of LBD display Aβ plaques in the MTL [52]; diversity in the Aβ aggregates may even underlie progression of disease into AD versus LBD as it has been observed that Aβ aggregates in LBD are comprised mainly of the Aβ1–42 peptide and the ratio Aβ1–42 to Aβ1–40 peptides is higher than in AD [61, 63]. AD/ALB may represent off-pathway, attenuated LB pathology induced by an Aβ strain more specific for tau misfolding; conversely LBD is the opposite with its own attenuated form of misfolded tau but more aggressive misfolded αsyn aggregates (Fig. 11). Although concurrent tau pathology is less common than Aβ in diffuse LBD [52], co-fibrils containing both αsyn and tau have been shown to occur which may have unique prion-like properties and could themselves modulate pathologic progression [32]. Interactions between proteins implicated in neurodegeneration are receiving increasing study due to their possible role in promoting strain diversity amongst the diseases, and the findings herein reinforce that such pathologic interactions are primed to occur in the amygdala where ample co-pathologies are present amongst the spectrum of neurodegenerative diseases [51, 73].

The extensive presence of specific post-translational modifications of αsyn in the amygdala and MTL is another modality by which αsyn in the amygdala could be converted into a more potent form. Biochemical studies revealed that the MTL/amygdala harbored a larger amount of high molecular weight αsyn compared with the cingulate cortex; these bands may represent ubiquinated or oligomerized forms of αsyn. In addition, the presence of 2 major carboxy-truncated forms of αsyn within the MTL/amygdala but not the cingulate cortex may be of importance. Regional specificity in calpain and cathepsin expression and activity may underlie the abundance of these truncation products in the amygdala compared with other regions [28, 82]. In a previous examination of a single case of familial PD/LBD, the localized detection of major truncated αsyn products mainly to the amygdala was also observed which is in line with our own results [55]. Truncated forms of αsyn are known to alter the prion-like and aggregative properties of αsyn [93, 99], and their presence in the amygdala may contribute to unique neuropil pathologies likely as a component of αsyn fibrils also comprised of FL αsyn and other post-translationally modified αsyn proteins. In particular, the very early involvement of the amygdala in LBD and/or cases where only the amygdala has αsyn pathology suggests that the amygdala has the capacity for initial αsyn aggregation and not always necessarily prion-like spread. These differences in prion-like activity could in part be due to the large increase in aggregation propensity truncation of the C-terminus imparts to αsyn.

The amygdala is often considered to be a loosely related collection of separate nuclei with different functions [73], and although regional variation in αsyn pathology was observed within the amygdalas the exact nuclei involved were not distinguished; it has been previously noted that the central and accessory cortical nuclei are the most heavily afflicted in advanced PD [9]. Never the less, the findings of this study agree with an emerging theory that the amygdala is critical in either the initiation of aggregation, or the conversion of pathologic protein aggregates into more potent forms [73]. In addition to cell autonomous factors such as neuro-inflammation and impaired lysosomal autophagy [6, 106], the diversity of pathologic αsyn species occurring within the amygdala due to conformational changes, post-translational modification, or templating from other misfolded proteins such as Aβ should be a key target of study to understand why αsyn pathology may rapidly progress (LBD) or stay localized (AD/ALB). Future studies should utilize appropriate combinations of retrieval techniques and antibodies to maximize detection of neuropil pathology that is seemingly more relevant to disease, and incorporate assays to measure prion-like properties of αsyn within a given brain region to determine which areas of the brain would serve as suitable targets of disease modifying therapeutics designed to halt prion-like spread of pathology.

## Conclusions

In summary, the findings of this study first demonstrated that the presence of an aggressive “strain” of αsyn as is the case with A53T αsyn in familial disease is sufficient to start a pathologic cascade incorporating WT naive αsyn. Furthermore, pathologic determinants of progression from isolated LBs within the amygdala to diffuse synucleinopathy were studied which indicated that thread-like neuropil αsyn pathologies including astrocytic αsyn aggregates are more relevant in predicting widespread pathology than the presence of LBs. The amygdala, due to its early involvement in multiple neurodegenerative diseases and unique pathologies discussed herein, is a key location where diverse strains of misfolded αsyn resulting in detrimental neuropil aggregates are likely to occur.

## Additional file

Additional file 1: **Figure S1.** Quantitation of LRP in LBD brain regions across a panel of antibodies individual cases. Three areas of dense pathology within the cingulate (C) or amygdala (A) of 9 LBD cases stained with 3 different antibodies without or with (FA) retrieval were subject to positive pixel analysis; average positivity and error bars (std) are displayed for each case, region, and antibody without or with FA. Without FA retrieval, all antibodies detect similar amounts of LRP within the amygdala versus the cingulate cortex; with FA retrieval a large increase in labeled amygdala pathology is evident with all antibodies whereas a lesser increase in pathology is seen in the cingulate cortex and only with antibody 94-3A10. With FA retrieval, the average amygdala pathology burden is significantly greater than the cingulate cortex for all antibodies. (TIF 4434 kb)

## Data Availability

All data generated and analyzed during this study are included in this published article and its supplementary information files.

## Abbreviations

AD: Alzheimer’s disease
AD/ALB: Alzheimer’s disease with amygdala restricted Lewy bodies
APOE: Apolipoprotein E
BCA: Bicinchoninic acid assay
BSA: Bovine serum albumin
CAA: Cerebral amyloid angiopathy
FA: Formic acid
FBS: Fetal bovine serum
FL: Full-length
FTLD-TDP: frontotemporal lobar degeneration with TAR DNA-binding protein 43 inclusions
GFAP: Glial fibrillary acidic protein
HS: High-salt
iLBD: incidental Lewy body disease
LB: Lewy body
LBD: Lewy body dementia
LNs: Lewy neurites
LRP: Lewy related pathology
MTL: Medial temporal lobe
PBS: Phosphate buffered saline
PD: Parkinson’s disease
PSP: Progressive supranuclear palsy
RIPA: Radioimmunoprecipitation assay
SNpc: Substantia nigra pars compacta
TBS: Tris buffered saline
WT: Wild type
αsyn: α-synuclein
