# Supplementary figures and images for: Unique α-synuclein pathology within the amygdala in Lewy body dementia: implications for disease initiation and progression
Source: Acta Neuropathol Commun. 2019 Sep 2;7:142. doi: 10.1186/s40478-019-0787-2 (PMC6718048; doi:10.1186/s40478-019-0787-2)

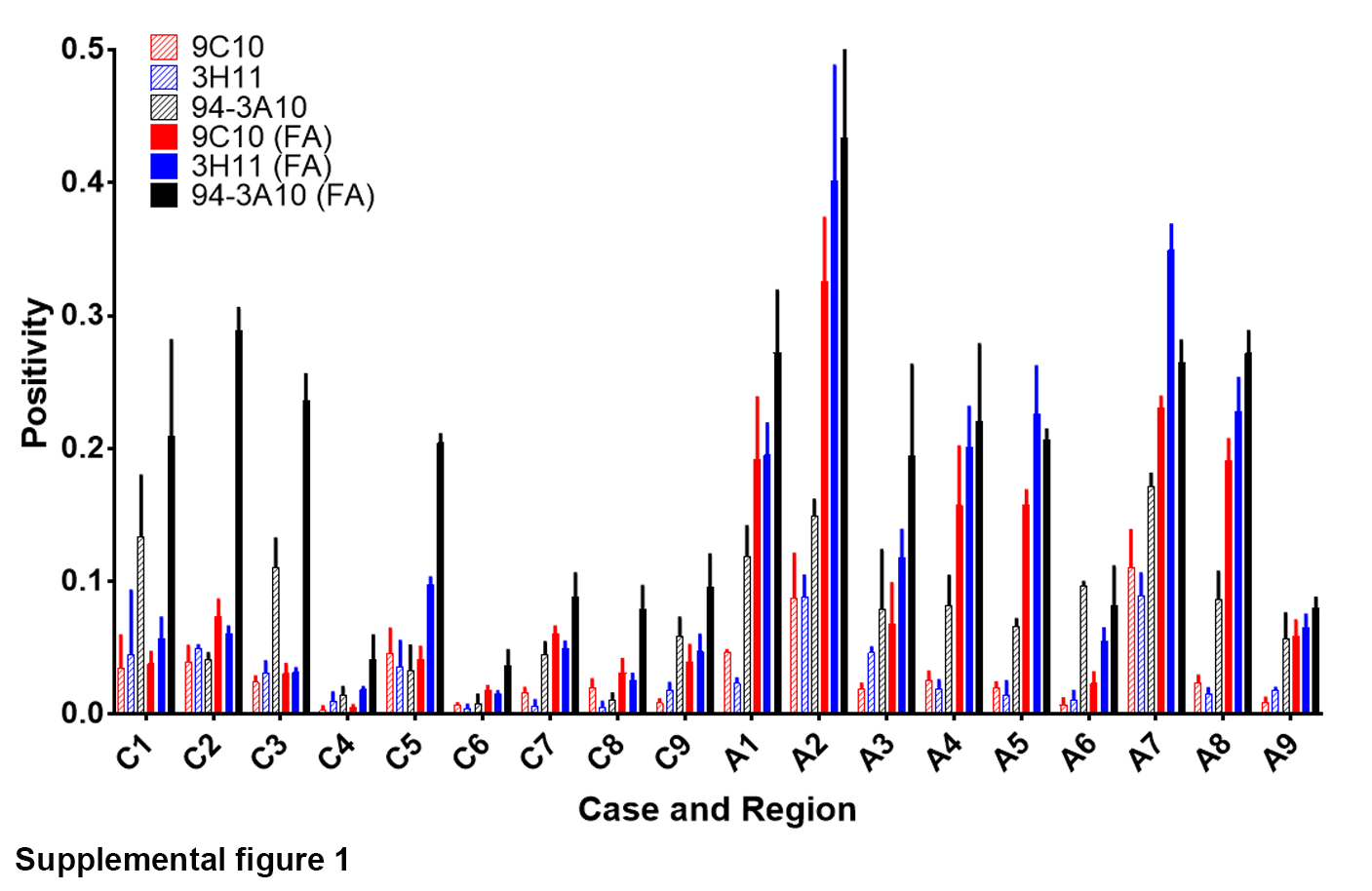

Supplement: Supplementary file 1 — Figure S1. Quantitation of LRP in LBD brain regions across a panel of antibodies individual cases. Three areas of dense pathology within the cingulate (C) or amygdala (A) of 9 LBD cases stained with 3 different antibodies without or with (FA) retrieval were subject to positive pixel analysis; average positivity and error bars (std) are displayed for each case, region, and antibody without or with FA. Without FA retrieval, all antibodies detect similar amounts of LRP within the amygdala versus the cingulate cortex; with FA retrieval a large increase in labeled amygdala pathology is evident with all antibodies whereas a lesser increase in pathology is seen in the cingulate cortex and only with antibody 94-3A10. With FA retrieval, the average amygdala pathology burden is significantly greater than the cingulate cortex for all antibodies. (TIF 4434 kb) [file 40478_2019_787_MOESM1_ESM.tif]
